# A Case Report of a Sacral Giant Cell Tumor Treated With Block Resection and Lumbo-Pelvic Fixation

**DOI:** 10.7759/cureus.31224

**Published:** 2022-11-07

**Authors:** Carlos L Hernandez, Salvador M Díaz, Renat Nurmukhametov, Evgeny Goncharov, Manuel de Jesus Encarnacion Ramirez, Ismail Bozkurt, Issael Jesus Ramirez Pena

**Affiliations:** 1 Spinal Surgery, Misericordia Clinic, Barranquilla, COL; 2 Clinical and Translational Research, La Misericordia Clínica Internacional, Barranquilla, COL; 3 Neurosurgery, Russian People's Friendship University, Moscow, RUS; 4 Central Clinical Hospital of the Russian Academy of Sciences, Traumatology and Orthopedics Center, Moscow, RUS; 5 Neurological Surgery, Peoples friendship University of Russia, Moscow, RUS; 6 Neurosurgery, Cankiri State Hospital, Cankiri, TUR; 7 Neurooncology, Neurosurgery Oncology Fellow Royal Melbourne Hospital, Victoria, AUS

**Keywords:** lumbo-pelvic, spinal tumor, sacroiliac, giant cell tumor, partial sacrectomy

## Abstract

Giant cell tumors (GCT) are benign but locally aggressive neoplasms composed of osteoclast-like giant cells and fusiform to ovoid mononuclear stromal cells. GCT generally comprise 5-10% of all benign bone tumors; they appear most frequently between the second and third decades of life. These tumors are also distributed throughout the vertebral column. Approximately half of all spinal GTCs develop in the sacrum. Many cases remain clinically silent and are discovered incidentally during the study of minor trauma. Symptomatic tumors often mimic other common spinal pathologies. Imaging studies ideal for diagnosis are CT and MRI. The techniques used in the treatment of giant cell tumors are curettage or intralesional surgery, block resection, radiotherapy, and chemotherapy. Herein, we report on a 23-year-old female patient diagnosed with a tumoral mass in the anterior part of the sacrum, suggestive of GCT. The lesion was completely excised in two consecutive surgeries, and lumbopelvic fixation was performed with favorable immediate postoperative results. Careful surgical planning with a multi-disciplinary approach leading to block resection still remains the most viable option for the treatment of vertebral GCT.

## Introduction

Giant cell tumors (GCT) are rare and benign tumors of the bone. They are composed of osteoclast-like giant cells and fusiform to ovoid mononuclear stromal cells [1]. GCT generally comprise 5-10% of all benign bone tumors and are diagnosed most frequently between the second and fourth decades of life. They are more frequent in women than in men [2]. They are more frequently found in the epiphyseal region of long bones. The vertebral column is also a site for GCT; however, almost half of all spinal GCT are found in the sacrum [3]. Many cases remain clinically silent and are discovered incidentally during the study of minor trauma. Symptomatic tumors often mimic other spinal pathologies such as chordoma, meningioma, melanoma, and melanocytoma [4].

Imaging studies ideal for diagnosis are CT and MRI. CT shows an attenuation of the soft parts with defined margins and occasional sclerosis and osteolytic findings [3]. Variable signal strength with heterogeneous hypointense images is observed in T1- and T2-weighted MRI sequences [3]. The reinforcement pattern is heterogeneous, and necrosis can sometimes be found. The techniques used in the treatment of giant cell tumors are curettage or intralesional surgery, block resection, radiotherapy, and chemotherapy [1]. Herein, we report the results of a patient who underwent surgery for a sacral giant cell tumor with both anterior and posterior approaches, allowing for adequate exposure, block resection, and lumbo-pelvic fixation to avoid instability.

## Case presentation

A female patient of 23 years of age with no hereditary family history of importance and an unremarkable personal medical history up-to-date was referred to the department of neurosurgery. She had presented with pelvic pain emanating from the lumbo-sacral region, specifically at night. The pain radiated to the lower left limb, causing an alteration of the gait and a loss of the tip-toe gait. She had a left-sided foot drop and an unintentional weight loss of 14 kg in the last year.

**Figure 1:**
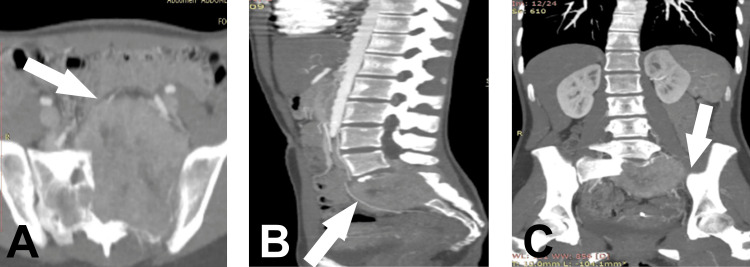
Contrast-enhanced lumbo-pelvic CT scan in (A) axial, (B) sagittal, and (C) coronal views. Note that the extensive tumoral mass (arrow) has obliterated the surrounding bony tissue, especially, the left sacroiliac joint with a prominent extension into the abdominal cavity.

**Figure 2:**
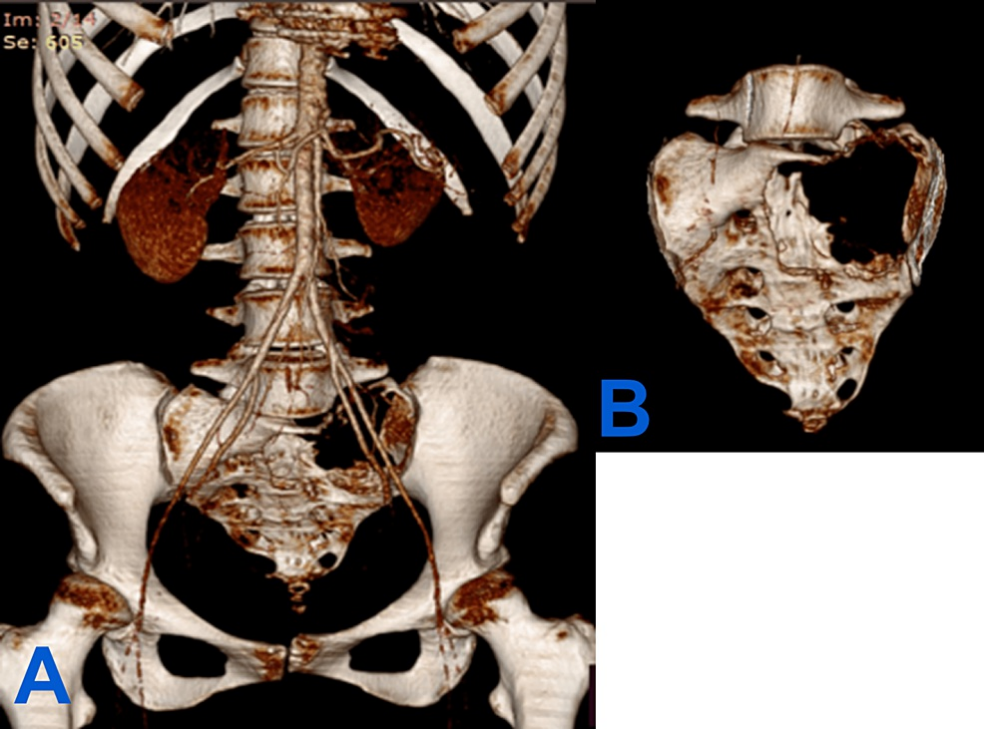
Preoperative planning with a 3D reconstruction of the CT. (A) Anterior view of lumbo-pelvic region with clear depiction of neighboring vital structures such as the internal and external iliac arteries along with what remains of the left sacro-iliac joint. (B) Anterior view of the sacrum showing the extent of the lesion allowing for correct planning of resection amount.

A pelvic ultrasound was performed, which revealed an intrapelvic mass. Imaging studies were completed with lumbo-sacral and pelvic CTs and MRIs. The CT revealed a tumoral mass in the sacrum predominantly on the left side, extending superiorly to the left L5 transverse process, laterally to the lateral sacral crest, and obliterating the S1 and S2 neural foraminae (Figure 1). The tumoral mass has partially defined edges and has invaded the left sacro-iliac joint with an osteolytic character. The lumbo-sacral canal invasion along with an extension to the paravertebral muscles can also be observed (Figure 2).

**Figure 3:**
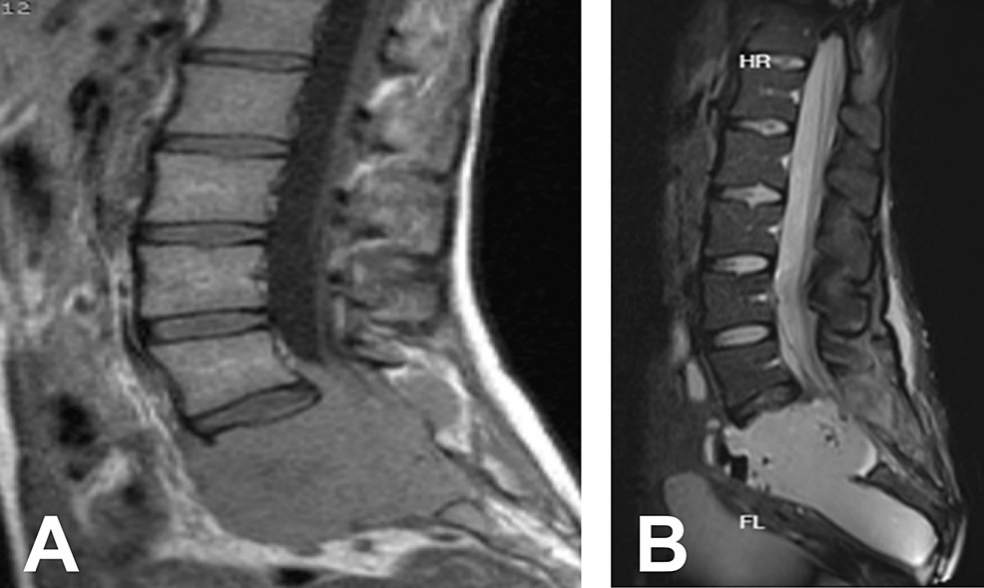
(A) Non-contrast-enhanced preoperative sagittal T1-weighted MRI; (B) postoperative MRI. Note the amount of resection and resection cavity.

An MRI was performed after the invasion of the spinal canal was confirmed; compression of the left L5, S1, and S2 roots and invasion of the left paraspinal muscles were observed (Figure 3).

A preoperative evaluation was performed that included a detailed medical history, a complete neurological examination, plain radiographic images, an MRI, and a CT with 3D reconstruction. Evaluation of preoperative gait status and bladder and bowel function was also done. She has no urinary or bowel dysfunction but has a gait disturbance due to the left-sided foot drop. Surgical excision was planned, and selective embolization of the tumor vessels 48 hours before surgery was performed by an interventional radiologist.

Surgical technique

The procedure was performed in two steps: an abdominal approach and a posterior lumbo-sacral approach. The abdominal approach performed by general surgery used an incision in the infraumbilical midline to the pubic symphysis, with mobilization of abdominopelvic viscera. Ligation of the large tumor vessels, followed by dissection of the margins, and a left anterior partial sacrectomy (with care not to injure the lumbosacral trunk or S1-S2 roots) were performed. Approximately 80% of the tumor mass was resected. The excision of the tumor was very laborious and with a significant blood loss (mostly from the tumor bed and the bone margins), requiring a transfusion of two units of erythrocyte suspension. The surgical margins of the anterior pathway were contaminated with tumor rupture and dispersion of the material.

After a week, the second surgical step was employed via a posterior lumbo-sacral approach. Through this approach, the remaining tumor was resected. A tumor mass was observed that invaded the left paraspinal muscles, which was resected with a wide surgical margin. A subperiosteal dissection of L3 to S3 was performed to clearly identify spinous processes. For partial sacrectomy, an S1 to S3 laminectomy was performed, and the S1-S2 roots were carefully identified and preserved. There was partial tearing of the dura mater, which was sutured. An L5-S1 discectomy was performed. The void space left, especially in the left sacroiliac joint, was filled with polymethylmethacrylate (PMMA). Bilateral transpedicular polyaxial screws were placed at the L4 and L5 levels. Two rods were placed, which were connected with a bar-to-bar connector to the screws placed in the iliac wing. There were no surgically sacrificed nerve roots.

**Figure 4:**
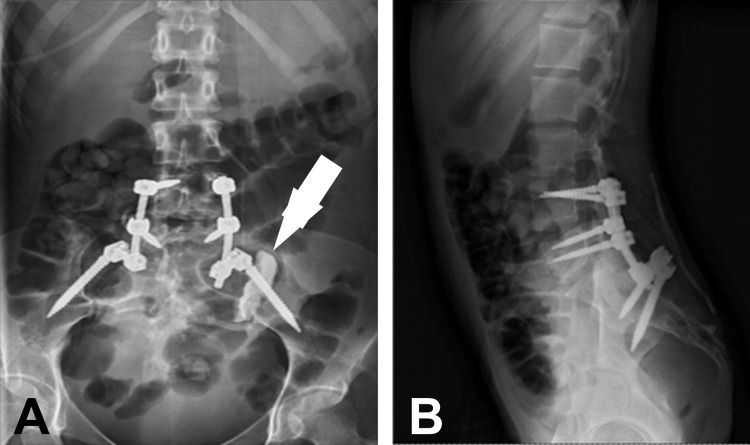
Simple radiography (A: anteroposterior, B: lateral) showing the fixation and placement of implants plus presence of bone cement (arrow) in surgical bed in the left sacroiliac joint.

In the immediate postoperative period of the second surgical period, the patient presented paresis in the extension of the knee and dorsiflexion of the left ankle, accompanied by hypoesthesia of the L5-S1 dermatome. Postoperative CT and X-ray analysis (Figure 4) showed the correct positioning of the instrumentation. Assisted ambulation was started 48 hours postoperatively. After discharge, the patient was referred to the oncology department and a home physiotherapy service. Histopathology was reported as the primary giant cell tumor of bone.

The patient has consented to her radiological images and results being published for medical use as long as they are anonymized.

## Discussion

The surgical treatment of sacral tumors is one of the great challenges for every spinal surgeon. The sacral canal and the pelvis are able to adapt to growing lesions, so sacrococcygeal tumors can be diagnosed after extensive growth [5]. Tumors of this region also have a latent period of specific symptoms such as radicular pain or urinary incontinence, which adds to the time of the first diagnosis. Surgical exposure of the sacrum is complex. The sacrum itself, when approached anteriorly, is seated deep within the pelvis behind critical neurovascular structures such as the sacral plexus and internal iliac artery and vein, along with pelvic organs such as the rectum, bladder, and uterus. The lateral approach is hindered by the iliac crests and lateral iliac joints [6]. The factors mentioned above render a standard, one-sided approach unfeasible. Multi-directional approaches and multi-disciplinary approaches should be tailored according to the needs of the patient and the characteristics of the tumor and surrounding anatomy. A tailored approach to the needs of the patient would allow for a more adequate approach, a greater excision amount, and the protection of healthy organs. The complexity of the anatomy of the pelvis and the presence of advanced disease mean that many times a multidisciplinary approach is required, in this particular case with the assistance of a general surgeon for the abdominal approach, along with an oncologist, physical therapist, and psychiatrist. This procedure is determined by numerous factors, such as the preoperative state of the patient, the anatomical characteristics of the lesion, the area of the sacrum that is compromised, and the biology of the tumor itself.

The need for careful preoperative planning and patient selection cannot be emphasized enough. Conventional radiological studies are usually insufficient in providing the detailed anatomical properties of the tumor and its surroundings. The surgeon must keep in mind that the oversized tumor may have displaced the normal positioning of anatomical structures and destroyed bony landmarks. Thus, all possible modern imaging technologies should be employed to prepare the surgeon for the upcoming challenge. The preoperative planning performed in a multi-disciplinary environment allows for the discussion of different approach strategies and the proper combination of techniques. Each surgical specialty has a different appreciation of the different anatomical structures located in the sacro-coccygeal region that prove beneficial in the treatment of the patient.

Total and partial sacrectomy present a high risk of morbidity leading to motor, urinary, bowel, and sexual dysfunctions. The extent of resection of the sacrum can also cause lumbo-pelvic instability. Thus, the amount of resection, determining the surgical borders, and the possibility of root sacrifice are vital in predicting the functional outcome of the patient [7]. The patient in this case benefited most from a left sacrectomy with wide surgical borders and the excision of the left sacroiliac joint, which warranted instrumentation to avoid instability. The level of midline sacrectomy correlates with the amount and degree of morbidity. As the sacrectomy level rises cranially, so do the incidence and degree of bladder and bowel dysfunctions, resulting in an increased hospital stay [8]. However, even in the presence of possible complications, in cases where local disease control cannot be maintained with other therapies such as intralesional surgery or adjuvant surgery or the possible clinical traits of the lesion warrant a total excision, the patient should be informed in detail about the possible benefits and risks of block resection. The proper surgical and oncological staging would allow for adequate and sufficient excision while minimizing the risk of complications. It should be noted that locally aggressive tumors require greater surgical excision for long-term disease control.

Giant cell tumors are benign but locally aggressive and highly recurrent lesions, with poor responses to radiation and chemotherapy. The oncological goal of surgery is total excision.

The broad resection of the tumor and the decompression of the neural elements associated with instrumented fusion allow for a stable spine, preserve or restore neural function, and prevent tumor recurrence [9]. This case allowed for sufficient sacrectomy and block resection while preserving sacral roots. The patient presented with neurological deficits postoperatively, most likely due to stretching and dissection of neural structures intraoperatively, but did fare better with physiotherapy. She did not present any bladder or bowel dysfunction, which is a vital marker for surgical complications.

The clinical presentation, the age of the patients, and the long-term surgical results make the reporting of complex cases treated by multidisciplinary teams extremely valuable. The broad resection of the tumor and the decompression of the neural elements associated with instrumented fusion allow for a stable spine, preserve or restore neural function, and prevent tumor recurrence. Tumor excision followed by fixation can reduce pain, decrease neurological deficits, and maintain or regain normal gait [10].

## Conclusions

The primary treatment of most sacral tumors remains surgical as chordoma, chondrosarcoma, and some giant cell tumors are resistant to radiation therapy and chemotherapy. Long-term control or even the cure of the disease can be achieved by a block resection of the tumor with sufficient margins. But due to the complexity of the sacro-coccygeal region and the need for lumbo-pelvic stability, a multi-disciplinary approach with meticulous preoperative planning is a must for sacral tumors. Even the most complex giant cell tumors of the sacrum can be successfully treated, but the patient should always be made aware of the possible complications and their implications for the remaining period of their life while including their views in the decision-making team.
